# Eating somewhere else: migrant food practices and provision in an informal settlement of Buenos Aires, Argentina

**DOI:** 10.3389/fnut.2026.1738744

**Published:** 2026-04-16

**Authors:** Maria Belen Fodde

**Affiliations:** The New School, New York, NY, United States

**Keywords:** food governance, food practices, food security, informal settlements, Latin America, migration

## Abstract

Informal settlements in Argentina are a destination for migrants looking for better living conditions. Migrating requires adapting to a new culture, including culinary traditions. This study introduces the case of Villa 20, one of the most populated informal settlements in Buenos Aires, where almost half of residents are migrants. The people who live in Villa 20 have developed strategies to access desirable meals that match their cultural preferences in precarious conditions. Their community networks are key to ensuring this, as they allow for the work done in soup kitchens and other community spaces. This is a mixed-method study that surveyed 177 households with the FAO Food Insecurity Experience Scale, and interviewed residents, government officials and local leaders. While findings suggest no significant differences in food insecurity levels between local and migrant communities, this work introduces the levels of food insecurity in the neighborhood, migrants lived experiences of food insecurity, and their coping strategies to maintain their culinary traditions. It also shows the role of migrant communities in the urban food systems of informal settlements, and the relevance of women in preserving migrant food practices.

## 1 Introduction

**Figure 1:**
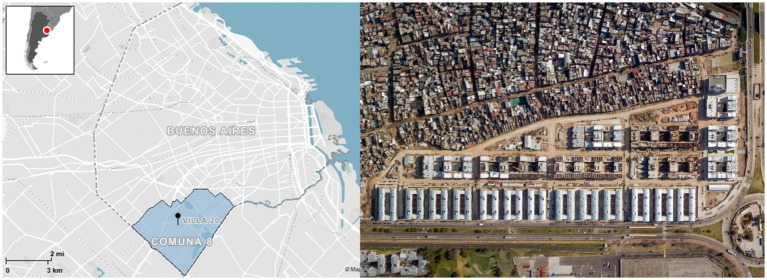
Left: Location and urban form of Villa 20, Buenos Aires. Source: Mapcreator.io (2025). Right: Reprinted with permission from Instituto de la Vivienda de la Ciudad de Buenos Aires (2020).

Villa 20 is a 30,000-resident informal neighborhood in the southwest area of the City of Buenos Aires, Argentina. It is one of the 49 informal settlements in the city (1) and one of the most populated. Like many Latin American informal settlements, it is characterized by poor-quality housing and basic services, as well as material deprivation resulting from the socioeconomic conditions of their residents (2). Most residents are low income and migrants that move there due to strategic location, affordable housing supply, and a community that hosts new neighbors through “bonds of family and loyalty” (3), 367. Villa 20’s first inhabitants settled before 1930, but the neighborhood grew significantly between 1940 and 1970 with the arrival of displaced families, including migrants from neighboring countries (4). Villa 20 is largely populated by migrant communities or *colectividades*, mainly from Bolivia, Paraguay, and Peru, whose traditions have shaped life in the neighborhood. The last census showed that 56 percent of its population was Argentinean, but a significant portion of older generations are foreign-born, as 41 percent of heads of household migrated from Bolivia (5) Figure 1.

More than 9,100 households live in Villa 20, and the last census estimated that 18 percent of workers relied on gigs, and that 24 percent of households were renters, while most of the remaining families did not have formal tenure (5). In relation to food access, at least 60 percent of households received government food assistance during the COVID-19 pandemic (6), a fragile situation that had been registered in a (7) survey where 74 percent of households responded running out of money to buy food in the previous 3 months (4).

The Food and Agriculture Organization (FAO) defines food security as “access to nutritious and sufficient food for all” (7), sustained over time (8). In addition to the quantity and the nutritional components of meals, food security should imply accessing a “decent meal” (9), desirable and satisfying to the consumer especially in exile, when it becomes a reference to the culture and place of origin (10). Existent literature has analyzed the consequences of migration in food practices, mentioning “culinary estrangement” as the challenge to acquire desired foods, as well as ‘social isolation’ that prevents them accessing food (11).

While there is literature focusing on processes of assimilation (as migrants adopt host cultures and lose their original one) including in food practices (12), other studies have focused on bidirectional dynamics such as alternation, acculturation, or at least segmented assimilation (13–17). One of the strategies to adapt to new locations and therefore maintain connections to places of origin is to intervene in the urban food systems, which are combinations of actors, processes and activities involved in the production, distribution, consumption and disposal of food (18). Understood as social-ecological systems, they combine governance and socio-ecological dynamics (including migration), among other elements (19). In informal settlements, food governance can be defined as polycentric (20), as access to food depends on three actors: the market, the State (through food programs), and local organizations. Local organizations are particularly relevant in informal settlements as they assist vulnerable communities distributing food and running soup kitchens (21, 22).

Informal settlements’ populations, including migrants, participate in the “informal” components of these food systems (23) to match culinary expectations and facilitate food access in a context of high deprivation (24). Informality is understood as an ambiguous nature that moves between legality and illegality (25), and previous studies have focused on the role that migrant, and sometimes transnational, communities play in shaping these economies (3, 26). Migrant communities consume but also shape their food systems as they participate in urban agriculture, establish restaurants that sell local dishes, and work as food vendors (27, 28).

There is a gender component to the participation of migrants in urban food systems. Studies show that many women migrating from neighboring countries are assisted by other women that had already migrated and settled before them, and do so to work on care duties and domestic work (29, 30). Care duties involve paid employment and participation in community soup kitchens (31). Women are, as in their households, in charge of providing food and feeding, balancing affordability and availability of resources to acquire what is needed (24, 32, 33) and applying a “practical knowledge” to do so (34).

Studies exploring migration and food security have analyzed the effects that remittances have on food insecurity levels (28, 35), while the migration-food insecurity nexus academic approach has found high prevalence rates among asylum seekers in South Africa (36), in Venezuelan refugee communities in Ecuador (37) and in urban Peru (38), as well as higher prevalence rates of food insecurity, with less diverse diets when comparing migrant to non-migrant communities in urban Namibia (39) and in Cape Town (28). Although achieving food security is one of the drivers to migrate, it is not always the result.

In Latin America, while there are studies applying the FAO Food Insecurity Experience Scale and other relevant tools (40, 41), there is limited research measuring food insecurity in informal settlements mostly due to data constraints. However, there are other examples in the Global South which show the potential of these studies (42–44). Additionally, there are limited research studies that holistically focus on the relationship between urban food systems and migration in informal contexts. Limited research is available on the lived experiences of food security and access faced by these communities in their new places (11), particularly in informal settlements. There is a need for research addressing the combination of food insecurity, informality, and migration, considering that emerging literature identifies migrants as vulnerable groups experiencing higher levels of food insecurity in comparison to local communities. Given the relevance of migration in Villa 20, and the existence of a solid network of social actors that contribute to food distribution (6), this study is a contribution to the analysis of food security and migration in Latin American and Global South informal settlements.

This article introduces findings of a study developed in 2023 to respond to the following research questions: 1. Do food insecurity levels vary by place of origin in informal settlements? Are migrant residents more food insecure than local residents?; 2. What are the factors that explain food insecurity from a polycentric food governance perspective?; 3. What are the strategies used by migrant communities to access sufficient, adequate, and decent meals? What role do they play in the urban food system?

## 2 Methods

This article introduces results of a mixed-method study conducted between March and July 2023^1^. Quantitative data included a survey of 177 households. The size of the sample was calculated using the G*Power software for 9,100 households (*α* = 0.05), which estimated the need for 138 responses to ensure statistical power. The sample was representative of the demographic (considering age groups and household size) and geographic (area where they lived) characteristics of households. The survey included questions on household composition, labor status, and connection to social actors of relevance (including whether they were beneficiaries of government programs or soup kitchens), and the FAO FIES module, a set of eight questions to analyze food insecurity in a population. Migration status of the respondents was determined by asking where they were born. The selection of respondents was randomized, as surveys were conducted on the street by the principal investigator and four research assistants who resided in the neighborhood. The analysis was done using R Studio and applying FIES methodology (45). Using the RM. Weights package, it was proved that the 8-item scale was accurate for infit (all values were between 0.7 and 1.3), outfit (values below 2) and reliability (0.79 value, with threshold being 0.7), Table 1.

**Table 1:** FAO FIES questions.

*In the last 12 months, was there a time when you or others in your household…*
1. Worried about not having enough food or whether the food in your home would end before you could buy, receive or produce more…
2. Were unable to eat healthy and nutritious food…
3. Ate only a few kinds of foods or were unable to eat certain kinds of food…
4. Ate less than you thought you should…
5. Had to skip a meal…
6. Ran out of food…
7. Felt hungry but did not eat…
8. Spent one entire day without eating…
*…because of a lack of money or other resources?*

**Figure 2:**
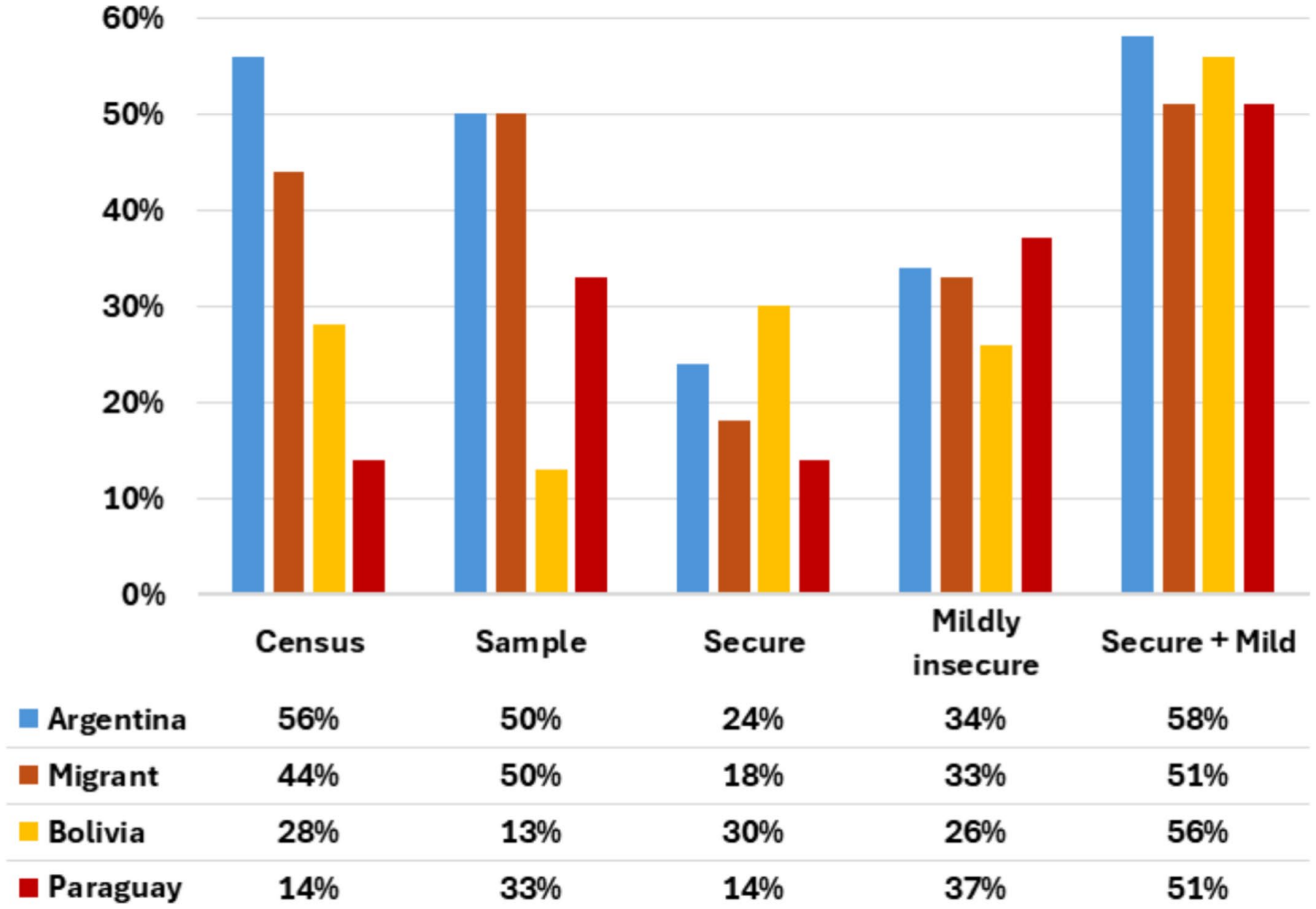
Sample distribution by country of origin, and food insecurity levels by place of origin.

While the scale used to collect data was validated, the raw score of positive responses to the FIES questions allows us to identify the level of food insecurity for each household, a useful estimation to analyze the general features of households in Villa 20 and their access to food. Households were classified by their experience of food insecurity following the thresholds established by FAO (question 4 between mild and moderate, and question 8 between moderate and severe) (45). While households with zero affirmative responses were considered food secure, households can be classified as mildly (1–3 affirmative responses), moderately (with 4–7 affirmative responses) or severely insecure (8 affirmative responses). Households were separated into two larger groups to ensure their comparability: those who are “more secure,” including food secure households and those with a mild level of insecurity (*n* = 96); and those who are “more insecure,” including those with a moderate or a severe level of insecurity (*n* = 81). Chi-square test of independence with a significance level of *p* ≤ 0.05 was used to assess if differences when comparing food insecurity levels across places of origin were statistically significant Figure 2.

Qualitative data included 24 semi-structured interviews with residents (*n* = 8), government officials (*n* = 6, in charge of food and social programs), local organizations or soup kitchens leaders (*n* = 8), and community members (*n* = 2), and observations of soup kitchens and public spaces. The sample ensured that relevant government programs, local leaders with different political affiliation and food distribution work, and households with different levels of food security and from different countries of origin were represented—although interviews with these subjects depended on their willingness to participate. The principal investigator conducted these interviews and analyzed them using Atlas. Ti software.

**Figure 3:**
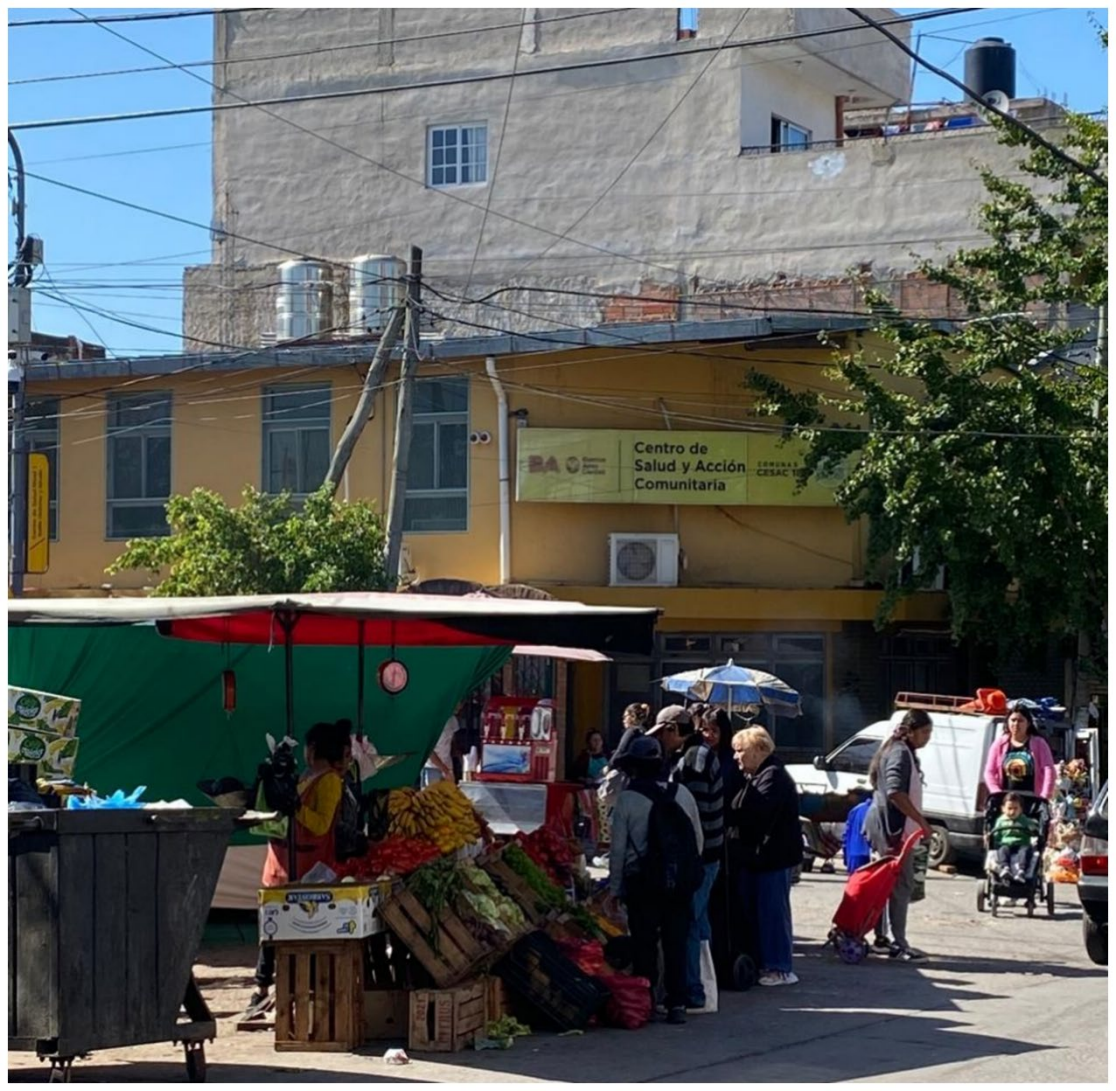
Villa 20 fair. April 2023. Source: author.

Safety concerns around entering and leaving the neighborhood were a limiting factor in data collection and limited reaching out to potential research subjects. Local research assistants ensured the collection of all necessary quantitative data Figure 3.

## 3 Results

The results revealed four significant findings related to migration, food security and food practices:

### 3.1 Migration

People move to Villa 20 for several reasons. Some of them mention affordability, since living in Villa 20 is cheaper than living in other areas of the city, and they can access jobs and services. Many migrate because relatives or acquaintances living in Villa 20 host them and share valuable information about adjusting to a new country: “they have a large network, and they share information (…) information travels super-fast. Communities act as networks, because the first person you call is your friend, your relative” (Interview with community member, June 2023).

**Figure 4:**
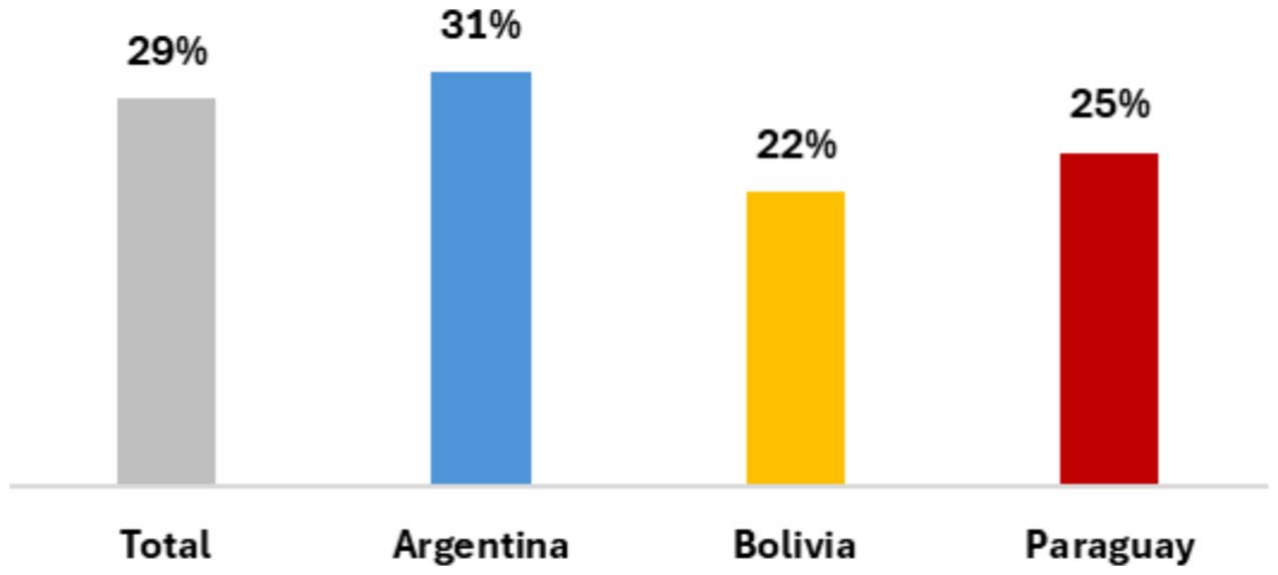
Local soup kitchen assistance by nationality.

However, a community member who worked and knows many families living in Villa 20 explains: “People did not choose to live here, it’s what they got, even if they are Argentinean or foreign migrants (…) as one of the guys that came from the countryside in Paraguay told me once, I asked him because he lived terrified, “why do not you go back?” And he told me: “You know what? In the countryside I used to eat worms, and here at least I eat. And maybe a bullet catches me, but I would not have lived anywhere else anyways” (Interview, June 2023). Other interviewees mention safety, lack of infrastructure and stigma as other challenges Figure 4.

### 3.2 Measuring food insecurity

While the census registered all Villa 20 residents, in this survey households were considered migrants based on the origin of the respondent. Only 50 percent of survey respondents were Argentineans, a smaller share than the 56 percent shown by the census. The survey underrepresented Bolivian residents (13 percent versus 28 percent) and overrepresented Paraguayans (33 percent versus 14 percent).

When analyzing the affirmative responses to the FIES questions 78 percent of households experienced at least some food insecurity. More than half of households surveyed, 56 percent, were either food secure or mildly insecure. When comparing migrant and local households, 57 percent of Argentinean and 51 percent of migrant households were secure or mildly food insecure. However, the chi-square test showed no statistically significant differences (*p* value = 0.41). Even though there were differences among migrant nationalities when exploring food insecurity (Bolivians had 56 percent of secure or mildly insecure households, while Paraguayans had 51 percent), differences were not statistically significant (*p* value = 0.6). Bolivian households had the largest group of households (30 percent) experiencing food security (zero affirmative answers to the FIES module) compared to Argentineans (24 percent) and Paraguayans (14 percent), Figure 2.

### 3.3 Government food programs

52 percent of households received government assistance through cash transfers. Ciudadania Porteña is a local program that distributes prepaid cards to buy food or hygiene products. Program eligibility depends on households’ composition and socioeconomic conditions (46). AUH-Alimentar, the Universal Child Allowance, is a federal conditional cash transfer program that aims to guarantee access to food, health and education for children under 19 years old whose parents are unemployed, informal, or domestic workers (47). For both programs, beneficiaries must be Argentineans or show proof of residency of at least 2 years. 48 percent of local households and 55 percent of migrant households received income from one of these programs (*p* value = 0.33).

In the case of AUH-Alimentar beneficiaries, 74 percent of households could not cover food expenses with the amounts distributed. This share grew to 88 percent for Ciudadania Porteña beneficiaries.

### 3.4 Uncovering migrant strategies in Villa 20

Besides their purchases in supermarkets outside the neighborhood, migrant communities consume and participate in their urban food system through three mechanisms: the local fair, a community garden, and soup kitchens.

The fair is a conglomeration of stalls and carts where people sell clothing, cooked food, products to cook, and work tools. They sell staples to prepare each migrant community’s meal, such as pepper, *putaparió*, tapioca, potatoes, and onions. Families buy prepared meals at both the fair and restaurants in the neighborhood. Pablo, a resident, explains that “the neighborhood is very integrated to [migrant] communities, and at the fair is very common to see typical foods from Bolivia, from Peru. A *ceviche*… You have the option to eat it, same as *sopa de mani*, or a *chipa*, or a *bori bori*. All of that exists” (Interview, June 2023).

Another place to acquire herbs or vegetables for cooking is *Terrazas de Cultivo*, a community garden launched in 2017 managed by a group of migrant women. While their production is not massive, they grow herbs for themselves and for people that attend the garden Figure 3.

Finally, in 2023, there were 60 soup kitchens in Villa 20. Most of these were run by social or political local organizations, and some of them were funded by local or national government agencies. Of families surveyed, 29 percent received food assistance from soup kitchens, either in the shape of cooked meals to eat on-site or to take home, or through the distribution of cooking staples. Some interviewees receive enough to feed their families daily, while others receive products less often. Gloria, born in Paraguay and living in Villa 20 for the past 20 years, says: “What I buy the most is cleaning supplies. The rest, thank God, I get at the soup kitchen, so I bring dinner. And we get some staples too. What I mostly buy is meat, cleaning supplies, and vegetables” (Interview, May 2023). Rosa, who migrated from Paraguay 15 years ago, says: “Sometimes at the soup kitchen we get products, sometimes one chicken, or two, or other staples, but not every day. And I get prepared meals, but I give them to my son who does not live with me” (Interview, May 2023).

Argentineans received the largest share of assistance from organizations (31 percent), followed by Paraguayans (25 percent) and Bolivians (22 percent). Each migrant community has its own network of soup kitchens, and people living in Villa 20 mention discrimination between nationalities, in particular against Bolivian households who are perceived as receiving more assistance through government programs and organizations. Ana migrated from Paraguay and has received food from several soup kitchens. She explains: “Sometimes they treat you well, sometimes they do not. With me they cannot, because I defend myself. But with Bolivian women, they disparage them” (Interview, May 2023) Figure 4.

What soup kitchens distribute depends on the funding they receive from the local and national governments. Often, soup kitchen leaders develop strategies to improve the resources they have, either because they are not enough to meet the demand, or because they aren’t satisfactory. They try to make food more attractive, adding spices, onion, garlic to make it “less precarious” (Interview with soup kitchen leader, April 2023). They do this through their “acquired cooking knowledge,” as explained by Claudia, a soup kitchen leader: “We have a lot of acquired knowledge, each *compañera* has her own way of cooking, her traditions, there are a lot of communities here, Bolivian, Paraguayan” (Interview, April 2023). In all soup kitchens interviewed, most people cooking were women: “We are 9 or 10 people. Some of us come to cook and some others to clean. We only have one male peer, and then we are all women” (Interview with soup kitchen leader, April 2023). Maria, another leader says: “It has not occurred to me… that the one that always has to do… yes, always women” (Interview, April 2023). Local leaders explain that many cooks do not receive a salary for the work they do.

## 4 Discussion

The analysis of food insecurity showed that over three quarters of households experienced some level of food insecurity. However, it was not determined by place of origin. Contrary to existing studies that show that migrants are worse-off than local households, and that being an immigrant becomes an additional challenge to secure affordable, healthy, and culturally appropriate food, the survey included in this study showed no significant differences between these two groups. This quantitative result may indicate that challenges to achieving food security are common among people living in informal settlements, and being low-income, therefore, could explain food insecurity more than being a migrant household. Other socioeconomic determinants such as labor status, income, household size or education could be better determinants of food insecurity in these contexts (41).

Regarding governance factors that determine food access, the State, as a governance actor, has limited effects on guaranteeing food access and security for families living in informal settlements. While there are barriers to access these programs as “foreign nationals,” households manage to obtain this coverage, and place of birth of the respondent wasn’t a significant variable during the analysis. The issue, then, is the limited financial support to afford food. Even though government programs contribute to household budgets and food access, they also exclude families in need or provide insufficient financial support.

The role of local organizations as another governance actor helps to understand food access as well as strategies to ensure access to decent meals, as they allow migrants to continue their culinary traditions, fighting a process of direct assimilation (16). The setting where migration, cooking, and feeding have a stronger connection is in community soup kitchens, as they are social actors of relevance for feeding strategies for all families living in informal settlements, whether they are migrants or not. They contribute to household budgets and, in some cases, to other family members’ food baskets.

In addition to the community garden and the fair, soup kitchens are particularly relevant to migrant communities for several reasons. First, migrants arrive at soup kitchens through networks that share information about food access for their communities (3), fighting social isolation (11). Second, in Villa 20, as in most informal neighborhoods in Buenos Aires, soup kitchens benefit from the presence of migrant women, who are not only key to the migration process, but also cook in these kitchens and share their knowledge about food practices, applying their “acquired knowledge” to prepare meals that are desirable for their communities (48). Women feed their families and play the same role providing food to households relying on soup kitchens, juggling variables such as affordability, resource availability, and taste (9, 32, 34). They are significant actors in informal settlements’ food systems as they contribute to food access for all households, and they reinforce acculturation in migrant communities. The fact that most of this work is unpaid reinforces the disparity in care giving tasks and calls for policy responses that recognize community and care-giving work.

Finally, even though chi-square testing did not show statistically significant differences, migrant communities are not a monolithic group. Bolivians received less support from soup kitchens compared to Argentinean or Paraguayan households. Additionally, qualitative research showed that migrant communities prioritize their fellow compatriots and avoid distributing resources to people from other communities, which results in discrimination between groups (49), particularly against Bolivian households.

### 4.1 Limitations

While this study makes a significant contribution through measuring food insecurity and analyzing migrants’ food practices, a more detailed data collection process capturing nationality of all household members and their feeding experiences could enrich the analysis and policy recommendations for communities in informal settlements. Further research in other informal settlements should be developed to confirm results.

## 5 Conclusion

This study introduced a snapshot of the experience of migrating to an informal settlement and its impact on food security and food practices preservation. It showed that moving to a context of deprivation implies facing struggles related to food acquisition, translating to the levels of food insecurity. At the same time, migrant households intervene in their urban food system to ensure access to decent and culturally desirable meals. Research in Villa 20 shows the need to focus on the food systems of informal neighborhoods in general, as it can contribute to improving access to food for all residents, including migrant communities. In particular, it is necessary to do so as certain migrant groups are subjects of discrimination and exclusion from certain networks that facilitate access to food. This case study shows the need as well for strengthening social protection systems through their food programs, considering the vulnerability of informal settlers. And finally, a holistic approach towards urban food systems requires the improvement of working conditions in these informal contexts, particularly in the case of women that develop tasks providing food.

## Data Availability

The datasets presented in this article are not readily available because none. Requests to access the datasets should be directed to Maria Belen Fodde, foddm724@newschool.edu.

## Footnotes

1 Project approved by BRANY SBER Institutional Review Board in March 2023. Protocol number 23–031-1244; Study title: “Food Governance and Food Security in Informal Settlements. The case of Villa 20 – Buenos Aires, Argentina”. IRB action: Exempt.

## References

[1] RENABAP. (2023). Observatorio de Barrios Populares. Accessed April 13, 2025. Available online at: https://www.argentina.gob.ar/obras-publicas/sisu/renabap/observatorio-de-barrios-populares

[2] Cravino MC. Las Villas de la Ciudad. Mercado e Informalidad Urbana. Los Polvorines: Universidad Nacional de General Sarmiento (2014).

[3] Gago V. Neoliberalism from Below. Durham, NC: Duke University Press (2017).

[4] OLA. (2020). "observatory on Latin America." Síntesis del Informe final “monitoring processes and outcomes in slum upgrading in Buenos Aires. Villa 20”. February 21. Available online at: https://observatorylatinamerica.org/pdf/NUP/Evaluacion/OLA_IVC_Villa20_SintesisInformeFinal_21feb2020.pdf (Accessed April 7, 2026).

[5] Instituto de Vivienda GCBA. Informe Final Censo 2016 Villa 20. Buenos Aires: IVC (2016).

[6] Diaz C María BF Carolina M Matías RD Daniela V. Gestionar la pandemia: dinámicas y estrategias entre el territorio y el Estado. El caso de Villa 20. Cuestión Urbana. (2020) 8–9:137–50.

[7] FAO, IFAD, UNICEF, WFP, and WHO. (2019). The State of Food Security and Nutrition in the World 2019. Safeguarding Against Economic Slowdowns and Downturns. Rome: FAO

[8] FAO. (2008). "An introduction to the basic concepts of food security." Food and Agriculture Organization of the United Nations. Accessed November 20, 2021. Available online at: https://www.fao.org/documents/card/en/c/2357d07c-b359-55d8-930a-13060cedd3e3/

[9] Garth H. Food in Cuba. The Pursuit of a Decent Meal. California: Stanford University Press (2020).

[10] de Certeau M Giard L Mayol P. The Practice of Everyday Life. Minneapolis: University of Minnesota Press (1998).

[11] Bakic Hayden T. Incomplete documentation, social isolation, and culinary estrangement: factors affecting food security among urban migrant populations in Mexico. Glob Food Secur. (2024) 42:1–6. doi: 10.1016/j.gfs.2024.100779

[12] Batis C Hernandez-Barrera L Barquera S Rivera J Popkin B. Food acculturation drives dietary differences among Mexicans, Mexican Americans, and non-Hispanic whites. The Journal of Nutrition Community and International Nutrition. (2011) 141:1906. doi: 10.3945/jn.111.141473, 21880951 PMC3174859 21880951

[13] Bowen S Hardison-Moody A Cordero Oceguera E Elliott S. Beyond dietary acculturation: how Latina immigrants navigate exclusionary systems to feed their families. Soc Probl. (2025) 72:819–40. doi: 10.1093/socpro/spad013

[14] Cleveland M Laroche M Pons F Kastoun R. Acculturation and consumption: textures of cultural adaptation Int J Intercult Relat. (2009) 33:212. doi: 10.1016/j.ijintrel.2008.12.008

[15] Portes A Fernandez-Kelly P Haller W. The adaptation of the immigrant second generation in America: theoretical overview and recent evidence. J Ethn Migr Stud. (2009) 35:1077–104. doi: 10.1080/13691830903006127, 23626483 23626483 PMC3634592

[16] Portes A Zhou M (1993) The new second generation: segmented assimilation and its variants 530 1 74 96 Ann Am Acad Pol Soc Sci: doi. 10.1177/0002716293530001006

[17] Sanou D O'Reilly E Ngnie-Teta I Batal M Mondain N Andrew C . Acculturation and nutritional health of immigrants in Canada: a scoping review. J Immigrant Minority Health. (2014) 16:24–34. doi: 10.1007/s10903-013-9823-7 PMC3895180 23595263

[18] UN Food Systems Summit. "Food Systems—Definition". In: Concept and Application for the UN Food Systems Summit. New York: Food Systems Summit (2021)

[19] Moragues-Faus A Zerbian T Lopez-Garcia D. A social-ecological approach to understanding urban food systems Ecol Soc. (2026) 31:20. doi: 10.5751/es-16516-310120

[20] Ostrom E. Beyond markets and states: polycentric governance of complex economic systems. Am Econ Rev. (2010) 100:672. doi: 10.1257/aer.100.3.641

[21] Merklen D. Pobres Ciudadanos. Las clases populares en la era democratica (Argentina 1983–2003). Buenos Aires: Gorla (2010).

[22] Mitchell A. Civil society organizations in the informal settlements of Buenos Aires: service providers and forces for change. International Society for Third-Sector Research. (2015) 27:37–59. doi: 10.1007/s11266-015-9561-7

[23] Crush J Ahmed Z. (2025). Considering the migration and food security Nexus in African cities. Waterloo: MiFOOD. Paper no. 39.

[24] Roy A-S Mazaniello-Chezol M Rueda-Martinez M Shafique S Adams A. Food systems determinants of nutritional health and wellbeing in urban informal settlements: a scoping review in LMICs. Soc Sci Med. (2023) 322:115804. doi: 10.1016/j.socscimed.2023.115804 36905724

[25] Roy A. Strangely familiar: planning and the worlds of insurgence and informality. Plan Theory. (2009) 8:7–11. doi: 10.1177/1473095208099294

[26] Portes A. The Economic Sociology of Immigration. New York: Russell Sage Foundation (1995).

[27] Bakic Hayden T Eguiguren M Alfaro Y. "Migration and urban food systems in Latin America". In: Bakic Hayden T Perez Martin J, editors. Urban Food Systems in Latin America. Territories, Mobilities and Governance. New York: Routledge (2025)

[28] Chikanda A Crush J Tawodzera G. Addressing Urban Food Security Challenges for South-South Migrants. Waterloo: MiFOOD Network (2024).

[29] Courtis C Pacecca MI. Genero y trayectoria migratoria: mujeres migrantes y trabajo domestico en el Area Metropolitana de Buenos Aires. Papeles de Poblacion (Universidad Autonoma del Estado de Mexico). (2010) 16:155–85.

[30] Mallimaci AI Magliano MJ. Mujeres migrantes sudamericanas y trabajo de cuidado en dos ciudades argentinas. Odisea Revista de Estudios Migratorios. (2018) 5:134. doi: 10.21670/ref.2320131

[31] Solans A Piaggio L. Cocina y comensalidad entre mujeres migrantes en Buenos Aires. Condiciones de vida y salud ConCienciaSocial Revista Digital de Trabajo Social. (2018) 2:90.

[32] DeVault ML. Feeding the Family. The Social Organization of Caring as Gendered Work. Chicago: The University of Chicago Press (1991).

[33] Pottier J. Anthropology of Food. The Social Dynamics of Food Security. Cambridge: Polity Press (1999).

[34] Boragnio A. "Entre comedores y bolsones, la situación alimentaria y las estrategias de acceso a los alimentos en las villas de la Ciudad de Buenos Aires". In: Tuñón I, editor. In La Cuestión Alimentaria en Tiempos de ASPO-COVID-19. Buenos Aires: Biblos (2023)

[35] Cassells D Costantini L Ashery AF Gadge S Pires DL Sánchez-Cortés MÁ . A 72h exploration of the co-evolution of food insecurity and international migration. arXiv preprint. arXiv:2407.03117. (2024). doi: 10.48550/arXiv.2407.03117

[36] Napier C Oldewage-Theron W Makhaye B. Predictors of food insecurity and coping strategies of women asylum seekers and refugees in Durban, South Africa. Agric Food Secur. (2018) 7:9. doi: 10.1186/s40066-018-0220-2

[37] Milan T Martens C. Venezuelan migration, COVID-19 and food (in)security in urban areas of Ecuador. Land. (2023) 12:1–12. doi: 10.3390/land12020517

[38] Hernandez-Vazquez A Vargas-Fernandez R Visconti-Lopez F Aparco JP. Prevalence and socioeconomic determinants of food insecurity among Venezuelan migrant and refugee urban households in Peru. Front Nutr. (2023) 10:1187221. doi: 10.3389/fnut.2023.1187221, 37396127 37396127 PMC10308025

[39] Kazembe L Tawodzera G Nickanor N. International Migration and the Urban Food Insecurity Nexus in Urban Namibia. New Directions in South-South Migration. Singapore: Springer (2025).

[40] Perez-Escamilla R. Can experience-based household food security scales help improve food security governance? Glob Food Sec. (2012) 1:120–5. doi: 10.1016/j.gfs.2012.10.006, 23795344 PMC3685197

[41] Smith M Kassa W Winters P. Assessing food insecurity in Latin America and the Caribbean using FAO'S food insecurity experience scale Food Policy. (2017), 71:48–61. doi: 10.1016/j.foodpol.2017.07.005

[42] Anand S Jagadeesh K Adelina C Koduganti J. Urban food insecurity and its determinants: a baseline study of Bengaluru Environ Urban 31 2 421 442. DOI. (2019). doi: 10.1177/0956247819861899

[43] Faye O Angela B Jane F Kanyiva M. Hunger and food insecurity in Nairobi's slums: an assessment using IRT models. J Urban Health. (2011) 88:S235–54. doi: 10.1007/s11524-010-9521-x 21234694 PMC3132228

[44] Joshi A Arora A Amadi-Mgbenka C Mittal N Sharma S Malhotra B . Burden of household food insecurity in urban slum settings. PLoS One. (2019) 14:1–24. doi: 10.1371/journal.pone.0214461 PMC6445475 30939157

[45] FAO elearning Academy. SDG Indicator 2.1.2—Using the Food Insecurity Experience Scale (FIES). Rome: FAO (2018).

[46] Gobierno de la Ciudad de Buenos Aires (2025). Ciudadanía Porteña. Accessed April 17, 2025. Available online at: https://buenosaires.gob.ar/desarrollohumanoyhabitat/ciudadania-Porteña

[47] Argentina R. (2025). Asignación universal por hijo (AUH). Accessed April 17, 2025. Available online at: https://www.argentina.gob.ar/justicia/derechofacil/leysimple/seguridad-social/asignacion-universal-por-hijo

[48] Solans A. Alimentación y mujeres migrantes en Buenos Aires, Argentina. Tradiciones, recreaciones y tensiones a la hora de comer. Rev Colomb Antropol. (2014) 50:119–39. doi: 10.22380/2539472x49

[49] Grimson A. Relatos de la diferencia y la igualdad. Los bolivianos en Buenos Aires. Nueva Soc. (1997) 147:96–107.

